# Effect of Turbulence Intensity on Cross-Injection Film Cooling at a Stepped or Smooth Endwall of a Gas Turbine Vane Passage

**DOI:** 10.1155/2014/256136

**Published:** 2014-01-28

**Authors:** Pey-Shey Wu, Shen-Ta Tsai, Yue-Hua Jhuo

**Affiliations:** Department of Mechanical and Automation Engineering, Da-Yeh University, 168 University Road, Dacun, Changhua 51591, Taiwan

## Abstract

This study is concerned with a film cooling technique applicable to the protection of the endwalls of a gas turbine vane. In the experiments, cross-injection coolant flow from two-row, paired, inclined holes with nonintersecting centerlines was utilized. The test model is a scaled two-half vane. The levels of turbulence intensity used in the experiments are T.I. = 1.8%, 7%, and 12%. Other parameters considered in the film cooling experiments include three inlet Reynolds numbers (9.20 × 10^4^, 1.24 × 10^5^, and 1.50 × 10^5^), three blowing ratios (0.5, 1.0, and 2.0), and three endwall conditions (smooth endwall and stepped endwall with forward-facing or backward-facing step). Thermochromic liquid crystal (TLC) technique with steady-state heat transfer experiments was used to obtain the whole-field film cooling effectiveness. Results show that, at low turbulence intensity, increasing Reynolds number decreases the effectiveness in most of the vane passage. There is no monotonic trend of influence by Reynolds number at high turbulence intensity. The effect of blowing ratio on the effectiveness has opposite trends at low and high turbulence levels. Increasing turbulent intensity decreases the effectiveness, especially near the inlet of the vane passage. With a stepped endwall, turbulence intensity has only mild effect on the film cooling effectiveness.

## 1. Introduction

The theory of Brayton cycle indicates that thermal efficiency of a gas turbine can be improved by increasing the turbine inlet temperature. The first stage vanes and blades of a gas turbine could be exposed to temperatures as high as 1900 K [1]. This has caused the turbine vanes and blades to work in much harsher environment than in the past. Film cooling technique is an effective way for the protection of these vanes and blades. In film cooling, coolant is injected from holes through the wall material with a certain injection angle. After exiting the holes, the coolant interacts with the main flow; thus, it may modify the main flow dynamics. The design purpose of a film cooling scheme is to form a low-temperature fluid layer which separates the wall from the hot gas stream so that the thermal load of the blade material can be reduced. How well the goal is achieved is determined by the interaction of the mainstream and the injected coolant. The cooling performance is usually quantified by film cooling effectiveness.

The complexity of a film cooling problem arises from two facts. First, the flow field near the endwall has complicated three-dimensional structure. In addition to the main flow, it contains secondary flows, such as horseshoe vortices, passage vortex, and corner vortex, and the interaction of these vortices [2–4]. Second, the problem is characterized by three temperatures:main flow temperature, wall temperature, and film coolant temperature. A complete description of the heat transfer and cooling characteristics should contain two parameters:heat transfer coefficient (HTC) and film cooling effectiveness. Numerous studies have been devoted to investigating various features of the problem. An excellent review can be referred to Simon and Piggush [5]. Only a few typical, related works are further noted as follows. Goldstein and Chen [6, 7] employed mass transfer technique to investigate film cooling effectiveness of a blade in the endwall region, with one-row [6] or two-row [7] film cooling holes and varying blowing ratio and density ratio. Comparing with two-dimensional region, their results showed little influence of the endwall on the pressure-side effectiveness. However, there was significant degradation of effectiveness in a triangle region on the suction surface, corresponding to the sweeping area of the passage vortex along that surface. The experimental results of Du et al. [8] using transient liquid crystal technique showed that wake turbulence increased the HTC while it decreased the film cooling effectiveness of a gas turbine blade. Hale et al. [1] used liquid crystal technique with steady-state experiment to measure the effectiveness distribution on a flat plate. They concluded that the effectiveness obtained from steady-state measurement was more accurate than that from transient measurement, except in the region very close to the holes. Dittmar et al. [9] measured distributions of the HTC and the effectiveness downstream of four different shaped film cooling holes using infrared thermography. Although there was superiority for each shaped hole, the fan-shaped holes with compound angles showed better effectiveness and lower HTC, especially at high blowing ratios. In the study of Wang et al. [10], three turbulence intensities (T.I. = 3%, 8.5%, and 18%) and five Reynolds numbers ranging from 2.4 × 10^5^ to 7.8 × 10^5^ were used to measure the HTC. The results were compared with that of a low turbulence case (T.I. = 0.2%). They concluded that the HTC on the suction surface had an obvious increase at high turbulence intensities because of earlier transition of boundary layer. The results of Ames [11–13] with four turbulence intensities (1%, 7.5%, 8%, and 12%) showed that turbulence length scale had significant influence on the HTC at stagnation zone and the pressure surface. Turbulence also decreased the effectiveness of shower-head film cooling.

The above-mentioned studies were conducted with smooth endwall condition. A stepped endwall may exist due to alignment of parts during installation or thermal expansion of materials during operation of the machine. Wu and Zhong [14] showed that a stepped endwall obviously affected the boundary layer development along the endwall and made the flow pattern in the vane passage even more complicated. Wu et al. [15, 16] showed that a forward-facing endwall step caused an increase in heat transfer coefficient while it caused a decrease in film cooling effectiveness at the endwall near the step. The passage vortex also climbed up the suction surface at an increased angle. Piggush and Simon [17] also found an increase in heat transfer at the leading edge of the platform with a forward-facing endwall step. The experimental results of Wu and Chang [18] at low main stream turbulence level (T.I. = 0.3%) showed that the film coolant coverage area was increased when the vortex of cross-injection coolant had the same sense of rotation as that of the passage vortex.

The present study focuses on cross-injection film cooling performance at the endwall of a gas turbine vane passage. The levels of turbulence intensity used in the experiments are T.I. = 1.8%, 7%, and 12%, which will be termed low, middle, and high T.I., hereafter, respectively. Three endwall conditions including smooth endwall and stepped endwalls with a forward-facing or a backward-facing step are considered. Other parameters in the film cooling experiments include three inlet Reynolds numbers (9.20 × 10^4^, 1.24 × 10^5^, and 1.50 × 10^5^) and three blowing ratios (*M* = *ρ*_2_ *u*_2_/*ρ*_*∞*_ *u*_*∞*_ = 0.5, 1, and 2, where the subscript 2 refers to the coolant property and the subscript ∞ refers to the main flow properties). Effects of parameters at varying turbulence intensity levels on the cross-injection cooling effectiveness are assessed from the observed results.

## 2. Experimental Setup

**Figure 1:**
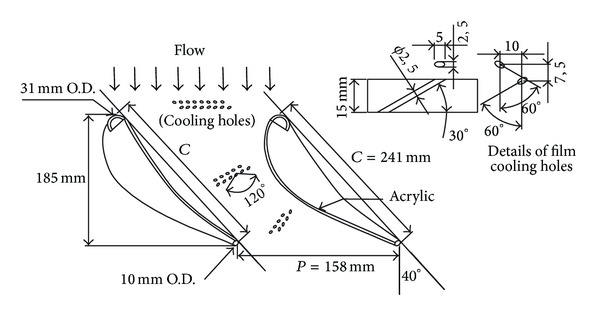
Two-half-vane test model and film cooling holes in the present study.

**Figure 2:**
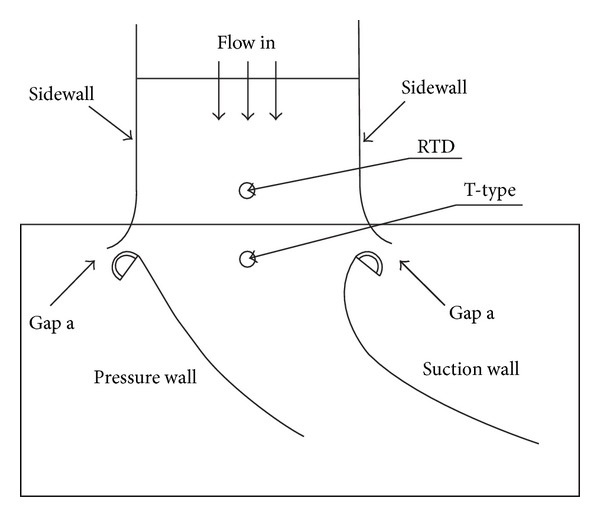
The test section with two side gaps and temperature measurement locations.

**Figure 3:**
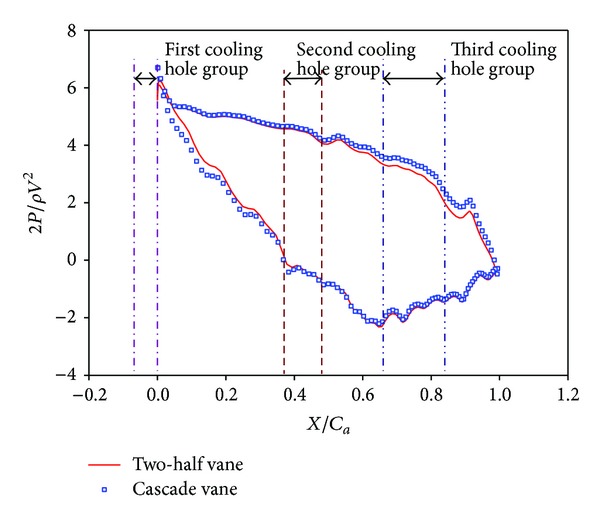
Comparison of the computed static pressure distributions for the two-half-vane model and a corresponding cascade.

The vane geometry of the present study is similar to that of Krishnamoorthy et al. [19]. The test model was scaled up by a factor of 3.46 for easier measurement. Instead of using a linear cascade, a two-half-vane acrylic model was used in the experiments, as shown in Figure 1. Two acrylic plates of 0.010 m thickness were heated and bent to the suction surface (SS) and the pressure surface (PS) profiles separately. Two acrylic bars of semicircular cross section 0.031 m and 0.010 m in diameter were attached to the ends of each plate to form the leading edge and the trailing edge, respectively, of the vane model. Both top and lower endwalls of the test model were made of acrylic plates of 0.015 m thickness. The lower endwall with film cooling holes is the investigated surface in the present study. Under the lower endwall is a plenum for settling coolant pressure before injection. The passage between the two-half vanes was used to simulate any flow passage in a turbine vane cascade. To achieve this similarity, side gaps (or bleed slots) between wind tunnel exit and the two vane leading edges must be provided to avoid channel flow situation, as shown in Figure 2. The gap sizes influence the saddle point, the pressure distribution, and the flow field in the vane passage. Therefore, suitable gap sizes must be determined. To this end, the CFD package FLUENT was used to compute the velocity and pressure fields for the two-half-vane model and for a cascade of the same vane size. Unstructured grids and two-dimensional, turbulent flow with k−*ε* model were employed in the computation. After several trials, appropriate gap size (0.009 m) was chosen so that the pressure distributions on the vane surfaces for both half-vane and cascade models had a reasonably good match (see Figure 3).

The film cooling holes at the lower endwall are divided into three groups, as also shown in Figure 1. Each group has two rows. All the coolant holes are of cylindrical type and have an inclination angle of 30 degrees with the endwall surface. In each group of the film cooling holes, both the first-row holes and the second-row holes have an orientation angle of 60 degrees, but they have opposite senses of rotation, with respect to the centerline of the passage. Therefore, each pair of the coolant holes, one from the first row and the other from the second row of corresponding count number, forms an included angle of 120 degrees. The coolant from paired holes forms a cross-injection pattern with nonintersecting centerlines. The key idea of the cross-injection design is the following. Because the extended centerlines of the paired holes do not have an intersection, the shearing of the injected jets, if they interact, will induce a vortex flow which tends to gather the coolant and prevent it from spreading into the main flow quickly. When the coverage area of the coolant on the endwall surface is increased, the film cooling performance will be better.

**Figure 4:**
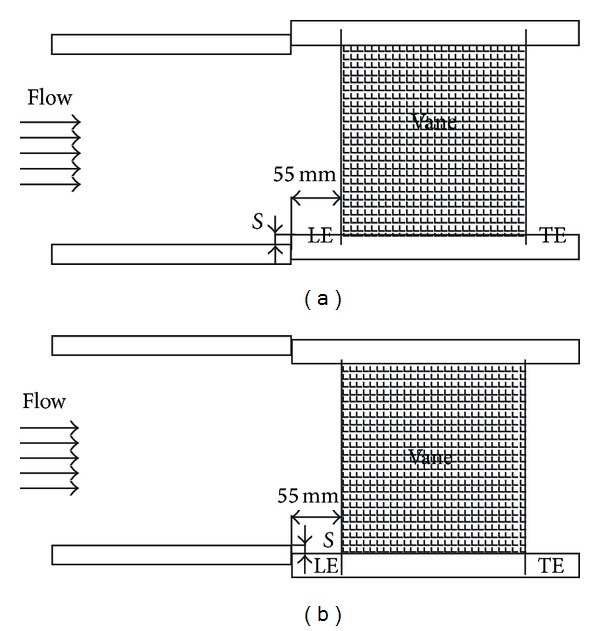
Stepped lower endwalls. (a) Forward-facing step. (b) Backward-facing step.

The test model was placed on four jacks that were used to adjust the vertical position of the model relative to the exit of the wind tunnel lower wall to simulate the stepped and smooth endwall conditions. For the case with a smooth endwall, the wind tunnel exit and the endwalls of the vane model were flash mounted so that the boundary layer continued to develop along the endwalls. For stepped endwall, the step (*S*), either forward facing or backward facing, occurs at 0.055 m upstream of the leading edge plane of the vane model, as shown in Figure 4. From a measurement of the allowable displacement of the seal and the vane dimension in a real turbine at room temperature, a reasonable step size is arbitrarily chosen as 4% of the true chord length (*C*); namely, *S*/*C* = +4% for a forward-facing step and *S*/*C* = −4% for a backward-facing step.

The main flow in the experiment is provided by a blowing type wind tunnel with fan power of 5.7 kW. A high-power air heater following the fan has a PID feedback control using the measured temperature of an RTD (Figure 2) for setting main flow temperature and heating speed. The hot air is then fed into a tunnel of cross section 0.306 m × 0.306 m. It enters a settling chamber and goes through a series of bar grids and fine screens for mixing. No additional parts for turbulence enhancement were installed for the case with low turbulence intensity (T.I. = 1.8%). For middle and high turbulence intensities (T.I. = 7% and 12%), another bar grid (with blockage ratio BR = 0.36 and 0.52, resp.) was also installed after the flow straightening section and 1.43 C upstream of the vane leading edge. The bar grid consists of an array of 12 mm diameter horizontal wooden rods. For the case with T.I. = 7%, the rods are spaced by 31 mm, while for the case with T.I. = 12%, the rods are spaced by 22 mm, both measured from center to center. The end of the wind tunnel is a converging nozzle, which reduces the flow cross-sectional area to 0.200 m (*W*) × 0.306 m (*H*) at the exit, followed by a settling section of 0.200 m length with adjustable sidewalls. The shape of the converging nozzle is designed and fabricated with a sixth-degree polynomial. The above-mentioned test section was attached to the exit of the settling section. Distributions of local velocity and turbulence intensity were measured at this location with a hot wire anemometry and an *X-Y* table. Velocity nonuniformity was controlled to be within 1% except very near the corners of the tunnel. During the experiments, thermocouples were installed before the inlet of the vane passage and in the midspan location to measure the free stream temperature. The temperature distribution at the tunnel exit was uniform near the test endwall from measurement results.

The coolant flow was drawn from an air-conditioned room by an air compressor and adjusted by a pressure regulator. After flowing through an air dryer and an oil filter, the clean air went into a variable-area flow meter. A pressure tap and a T-type thermocouple were also installed upstream of the flow meter for the correction of density effect in the calculation of mass flow rate. After measuring the coolant flow rate, the air was fed into the settling plenum before it was injected into the mainstream. The settling plenum is an acrylic box attached to the bottom side of the test endwall. The plenum was designed to have three inlets with independent valves for flow rate control. Three thermocouples were located in the plenum to ensure even temperature distribution of the coolant in the plenum. The measured coolant mass flow rate, the cooling hole geometry, and the plenum conditions were used in the calculation of the blowing ratio.

## 3. Experimental Method and Data Reduction

Steady-state liquid crystal experimental method was used for measuring the film cooling effectiveness (*η*) which is related to the adiabatic wall temperature (*T*_aw_) through the relation

(1) $$\begin{array}{c} \eta =\frac{T_{\infty }-T_{\text{aw}}}{T_{\infty }-T_{2}}. \end{array}$$

The coolant temperature (*T*_2_) was measured in the coolant settling plenum. The free stream temperature (*T*_*∞*_) was measured at the passage inlet (Figure 2). In the present study, ranges of the measured temperatures are 29.4–32.1°C for the coolant temperature and 35.1–36.2°C for the free stream temperature. The effect of their density ratio on film cooling is negligible. To obtain the adiabatic wall temperature (*T*_aw_), a guard heater was attached to the bottom side of the endwall plate. The guard heater was covered by an insulation layer to prevent it from heating the coolant in the plenum. Surface thermocouples were installed on the upper and the bottom sides of the endwall plate at corresponding positions and away from coolant hole locations. A power supply and resistors were used to control the heating rates so that the surface temperatures on the upper and the bottom sides of the endwall plate at corresponding positions were the same (i.e., the adiabatic condition). When a steady state was established and the liquid crystal displayed clear color pattern, the image data and the temperature data were taken. The distribution of the adiabatic film cooling effectiveness was then calculated from (1). For better comparison of the film cooling performance, the effectiveness data was also averaged over passage width at every fixed axial position, *X*/*C*_*a*_ where *C*_*a*_ is the axial chord length. Thus the results obtained will be used for discussions in the next section.

**Figure 5:**
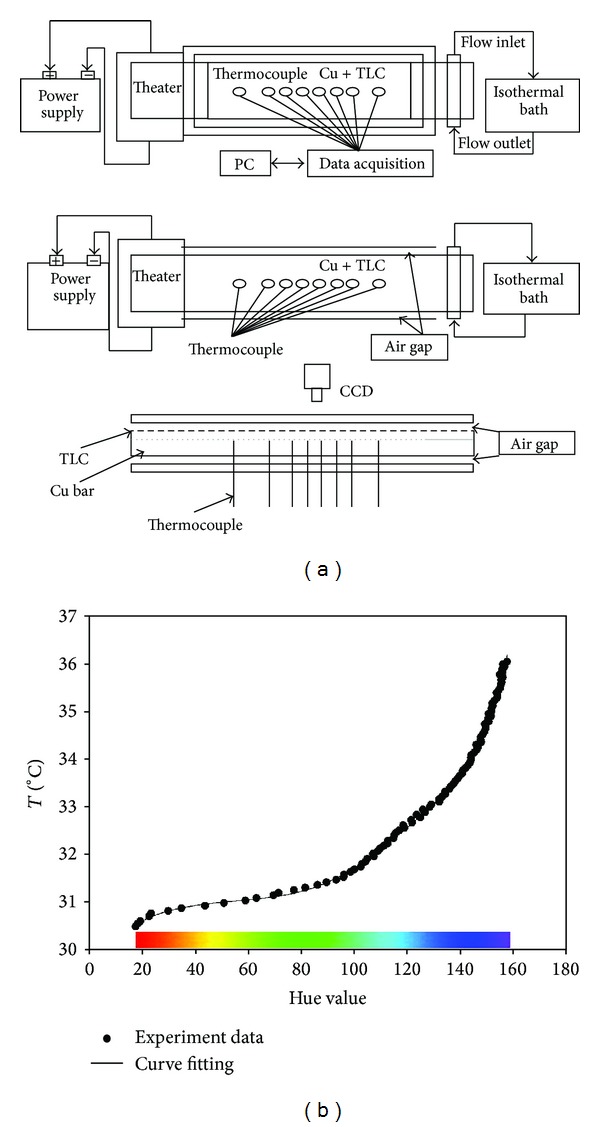
(a) Calibration setup and (b) *T-hue* relation of the R31C5W TLC.

The endwall (surface) temperature distribution was measured with an R31C5W (Hallcrest Co.) thermochromic liquid crystal (TLC). In all the experiments, liquid crystal technique with hue capture was used. The temperature to hue (*T-hue*) relation of the liquid crystal was calibrated under in situ optical conditions. For this purpose, one-dimensional heat conduction along a copper bar was established, and the measured temperature along the bar and the displayed TLC color pattern were recorded to obtain the *T-hue* relation. The calibration setup and the results are shown in Figure 5.

The free stream velocity and the turbulence intensity were measured at the passage inlet with a hot wire anemometer. The nonuniformity of the velocity distribution is less than 1.0% in the mainstream. The measured velocity was then used for the calculation of the inlet Reynolds number whose characteristic length was the chord length of the vane model. The turbulence intensity was calculated according to the following equation:

(2) $$\begin{array}{c} \text{T}.\text{I}.=\frac{\sqrt{{\overset{-}{u}}^{\prime 2}}}{\overset{-}{u}}=\frac{1}{\overset{-}{u}}\sqrt{\sum_{i=1}^{N}\frac{{\left(u_{i}-\overset{-}{u}\right)}^{2}}{N}}. \end{array}$$

In this study, thermocouples were calibrated using a high precision thermal well (HART SCIENTIFIC 9105) and a PT100 thermistor. The calibrated results ensure the accuracy of the temperature measurement to be within ±0.1°C. Estimation of experimental uncertainties was according to Moffat [20] and was based on 95% confidence level. The uncertainty of the Reynolds number was 3.5%. The uncertainty of the film cooling effectiveness from this experimental process was calculated to be 6.8%.

## 4. Results and Discussions

**Figure 6:**
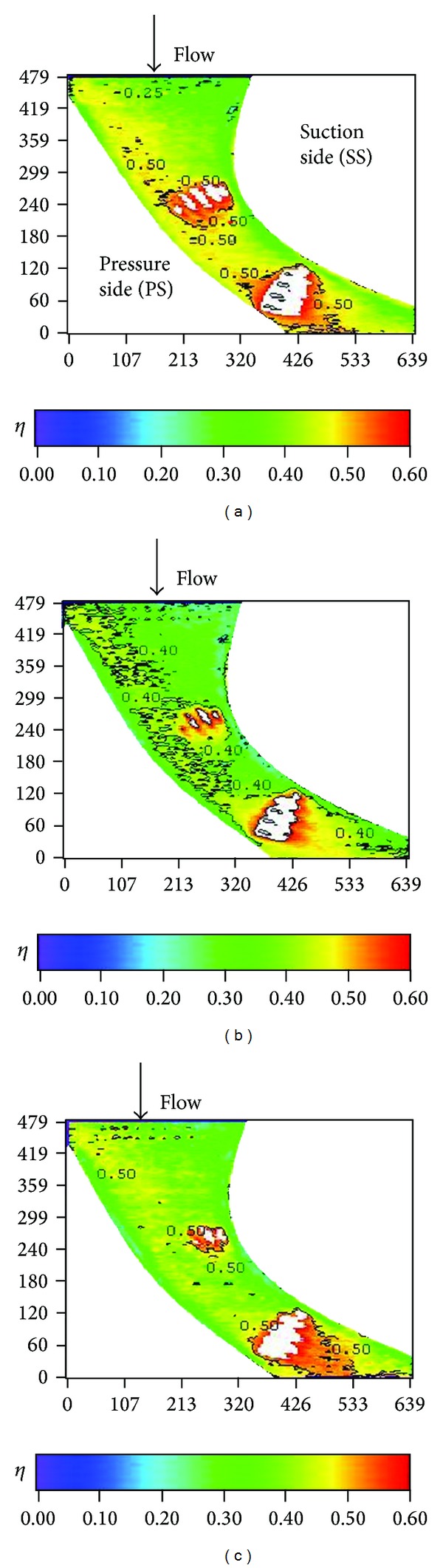
Distributions of local effectiveness at high turbulence intensity (*S*/*C* = 0%, *M* = 1, and T.I. = 12%). (a) *Re* = 92000. (b) *Re* = 124000. (c) *Re* = 150000.

**Figure 7:**
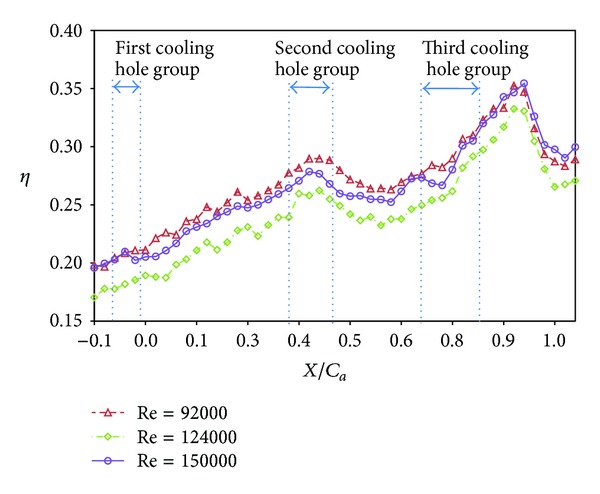
Effect of Reynolds number on averaged effectiveness at high turbulence intensity (*S*/*C* = 0%, *M* = 1, and T.I. = 12%).

**Figure 8:**
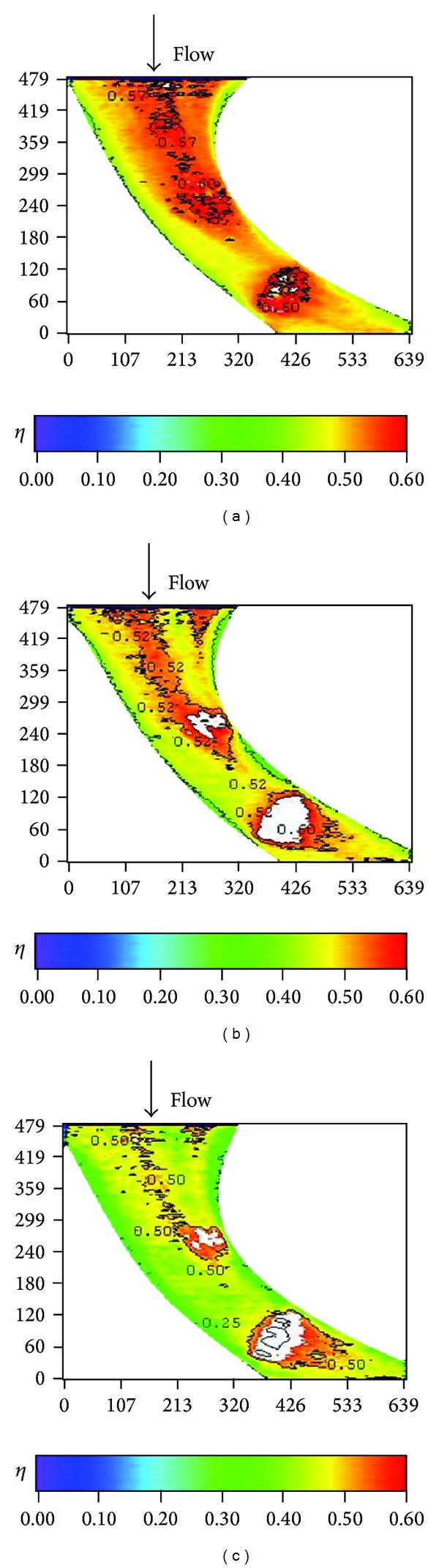
Distributions of local effectiveness at low turbulence intensity (*S*/*C* = 0%, *M* = 1, and T.I. = 1.8%). (a) *Re* = 92000. (b) *Re* = 124000. (c) *Re* = 150000.

**Figure 9:**
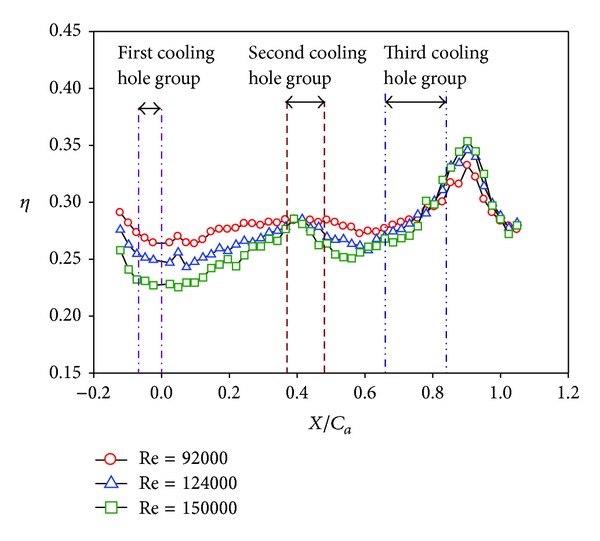
Effect of Reynolds number at low turbulence intensity (*S*/*C* = 0%, *M* = 1, and T.I. = 1.8%).

Distributions of local film cooling effectiveness and cross-stream averaged values are shown for nonstepped endwall condition at varying Reynolds number and two turbulence levels. Results with the high turbulence intensity (T.I. = 12%) are shown in Figures 6 and 7. The counterparts with the low turbulence intensity (T.I. = 1.8%) are shown in Figures 8 and 9. All the local distribution figures in this study, such as Figures 6 and 8, were reduced from wall temperature distribution pictures displayed by TLC and taken with a CCD camera of 640 (H) × 480 (V) resolution. Therefore, the unit for the horizontal and vertical axes is pixel for all of those figures. As can be seen from Figures 6–9, the highest effectiveness occurs at the end of the third cooling hole group, and the peak values in both figures are similar. The overall trend also shows an increase in the effectiveness along the axial position and a hump at the second and the third cooling hole group regions. This should be due to two reasons. The first reason is associated with the development of the endwall boundary layer. When the injected coolant interacts with the main flow, the boundary layer temperature changes, depending on how well the coolant stays attached to the endwall surface and the mixing of the coolant with the main flow. Normally, if the coolant does not separate from the endwall when leaving the hole, the effectiveness in the near-hole region has high values. This is the typical situation seen at the second and the third cooling hole group regions. Moving along the axial direction, mixing effect tends to increase the boundary layer temperature, hence, reduces the effectiveness downstream of the holes and results in a hump at the hole region. Because the three groups of cooling holes inject the same amount of coolant, and the third hole group is located downstream of the other two groups, the effectiveness at the third hole group is the highest. The second reason is that the third cooling hole group may be less influenced by the passage vortex. According to the flow visualization of [14], the passage vortex hits the suction surface near the half way through the passage. Less of the coolant would be swept away by the passage vortex. Figures 8 and 9 show that, at low turbulence intensity, increasing Reynolds number causes the effectiveness to decrease in most of the vane passage. The difference is reduced and the trend is even reversed at and after the third cooling hole group. However, at high turbulence intensity, Figure 7 shows no monotonic dependence of the effectiveness on the Reynolds number when the blowing ratio remains the same.

A remarkable difference between Figures 6-7 and Figures 8-9 exists in the region around the first cooling hole group. With highly turbulent flow, the film cooling effectiveness near the leading edge of the vane passage is drastically reduced. This is mainly due to the thinning of the boundary layer so that the injected coolant is quickly mixed with the main flow. The resulting effect is a dramatic suppression of the effectiveness at the passage inlet. As can be seen in later discussions, this phenomenon is common to all the combined effects of high turbulence level with other parameters.

**Figure 10:**
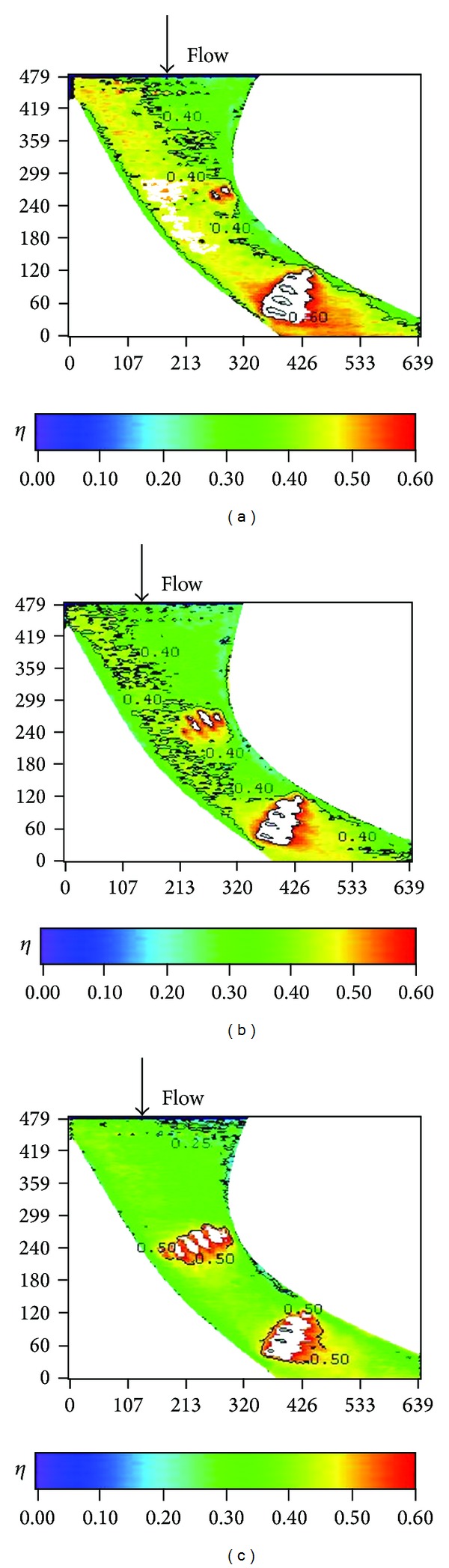
Effect of blowing ratio on local effectiveness at high turbulence intensity (*S*/*C* = 0%, *Re* = 124000, and T.I. = 12%). (a) *M* = 0.5. (b) *M* = 1. (c) *M* = 2.

**Figure 11:**
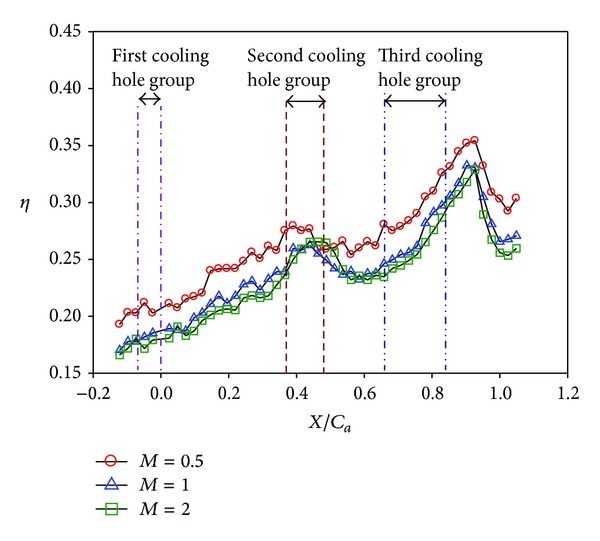
Effect of blowing ratio on averaged effectiveness at high turbulence intensity (*S*/*C* = 0%, *Re* = 124000, and T.I. = 12%).

**Figure 12:**
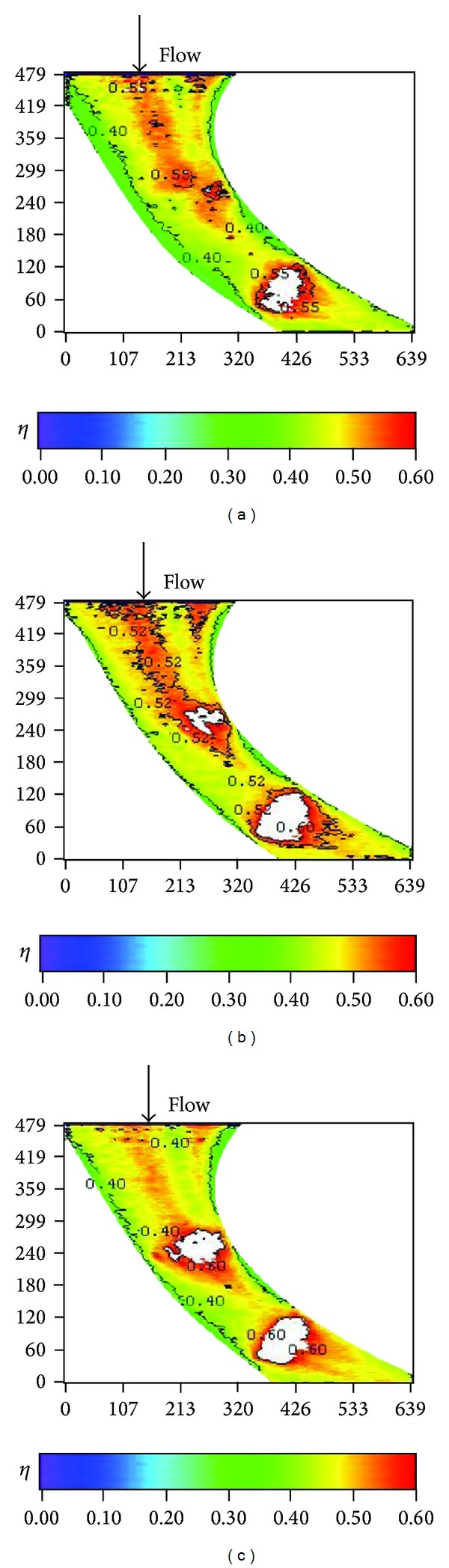
Effect of blowing ratio on local effectiveness at low turbulence intensity (*S*/*C* = 0%, *Re* = 124000, and T.I. = 1.8%). (a) *M* = 0.5. (b) *M* = 1. (c) *M* = 2.

**Figure 13:**
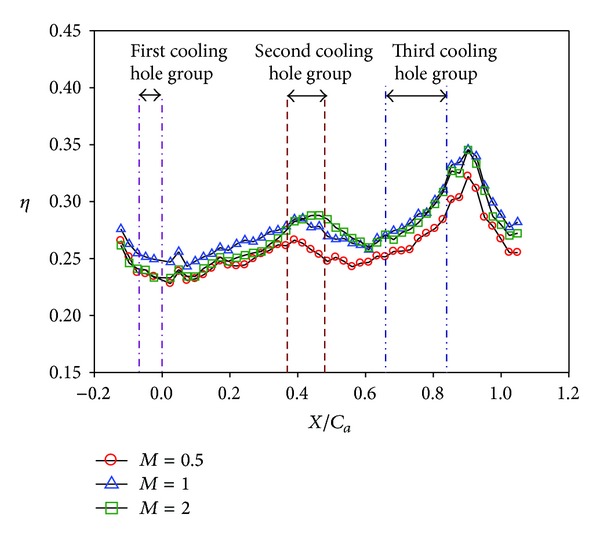
Effect of blowing ratio at low turbulence intensity (*S*/*C* = 0%, *Re* = 124000, and T.I. = 1.8%).

Effects of blowing ratio on the film cooling effectiveness for nonstepped endwall are shown in Figures 10, 11, 12, and 13 for high and low turbulence intensities, respectively. An obvious difference in the region surrounding the first cooling hole group is also seen in these figures. Again, this is due to thinning of the boundary layer at the passage inlet. In addition, these figures also show that the trend of the blowing ratio effect on the effectiveness is reversed from low turbulent flow cases to high turbulent flow cases. From observing the effectiveness pattern for the case with high turbulence and low blowing ratio, Figure 10(a), the cross flow along the endwall induced by the cross-stream pressure gradient increases the effectiveness of the endwall near the pressure surface (PS). This benefit is reduced when the blowing ratio is increased.

**Figure 14:**
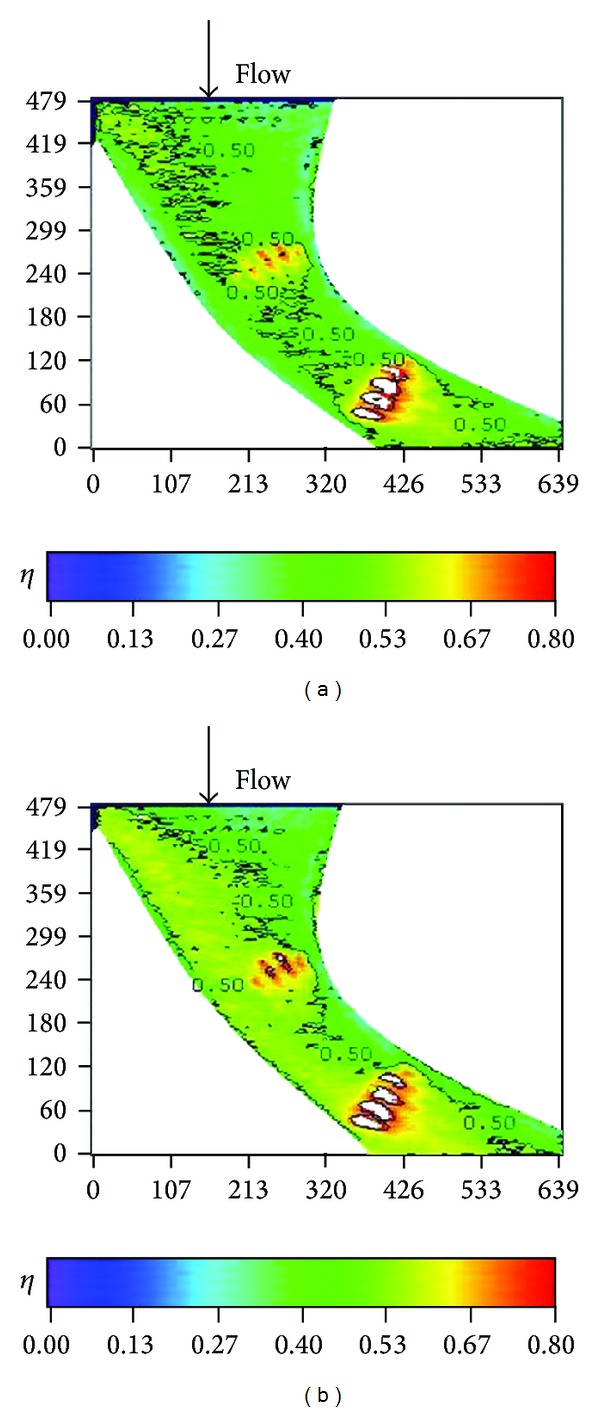
Effectiveness distributions on stepped endwall at high turbulence intensity (*Re* = 124000, *M* = 1, and T.I. = 12%). (a) *S*/*C* = 4%. (b) *S*/*C* = −4%.

**Figure 15:**
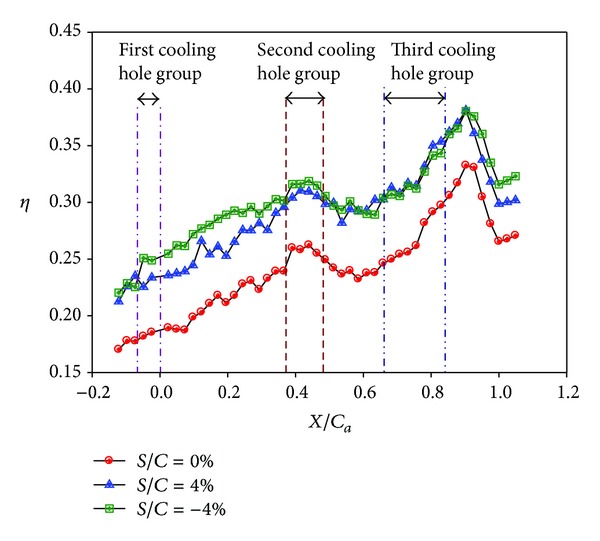
Effect of stepped endwall on averaged effectiveness at high turbulence intensity (*Re* = 124000, *M* = 1, and T.I. = 12%).

**Figure 16:**
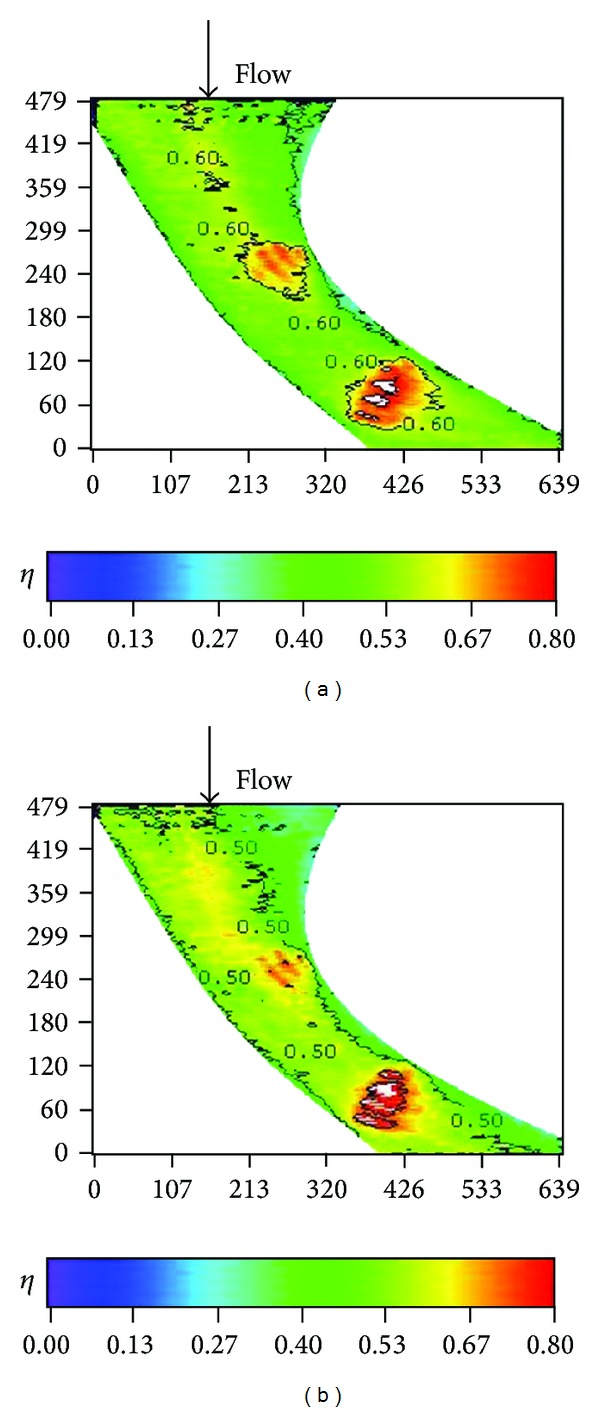
Effectiveness distributions on stepped endwall at low turbulence intensity (*Re* = 124000, *M* = 1, and T.I. = 1.8%). (a) *S*/*C* = 4%. (b) *S*/*C* = −4%.

**Figure 17:**
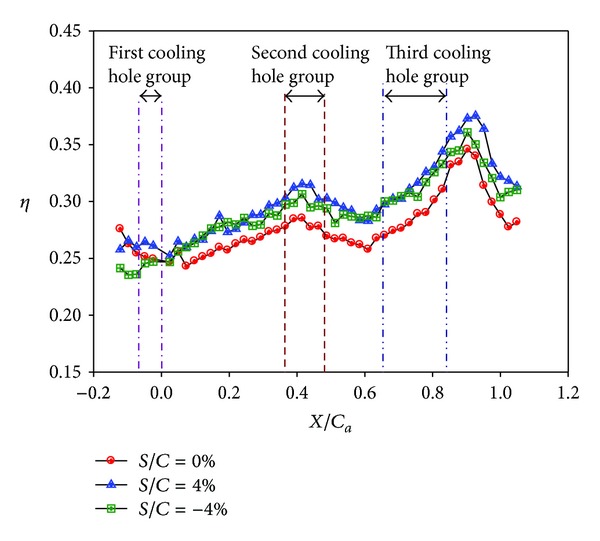
Effect of stepped endwall on averaged effectiveness at low turbulence intensity (*Re* = 124000, *M* = 1, and T.I. = 1.8%).

Figures 14–17 show the effects of the stepped endwall on effectiveness at high and low turbulence levels, respectively.  Figure 15 was obtained from averaging local effectiveness at each axial location in Figures 14 and 10(b), while Figure 17 was obtained from Figures 16 and 12(b). As mentioned above, an endwall step, if any, occurs 0.055 m (corresponding to *S*/*C*_*a*_ = − 0.23) upstream of the leading edge plane, as shown in Figure 4. Since the reattachment length increases from pressure side to suction side [14], the data shown in these figures include part of the reattachment region. Normally, the flow over a stepped endwall has separation and reattachment phenomena; the overall averaged effectiveness is higher than that of the nonstepped endwall case due to redevelopment of the boundary layer. However, for the low turbulence case with a backward-step endwall (*S*/*C* = −4%), flow reattachment at the first cooling hole group causes a local minimal of the effectiveness, and the value is lower than that of the nonstepped endwall case in that region. A comparison between Figures 15 and 17 shows that high turbulence level decreases the effectiveness around the first cooling hole group region, regardless of the endwall condition.

**Figure 18:**
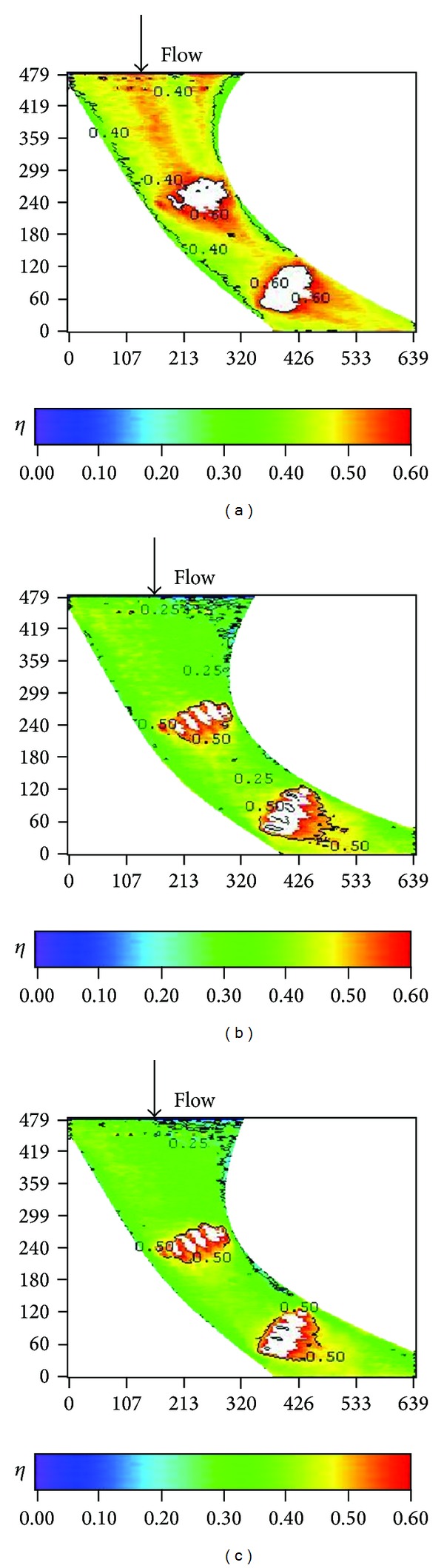
Effect of turbulence intensity on effectiveness distributions for nonstepped endwall (*Re* = 124000, *M* = 2, and *S*/*C* = 0%). (a) T.I. = 1.8%. (b) T.I. = 7%. (c) T.I. = 12%.

**Figure 19:**
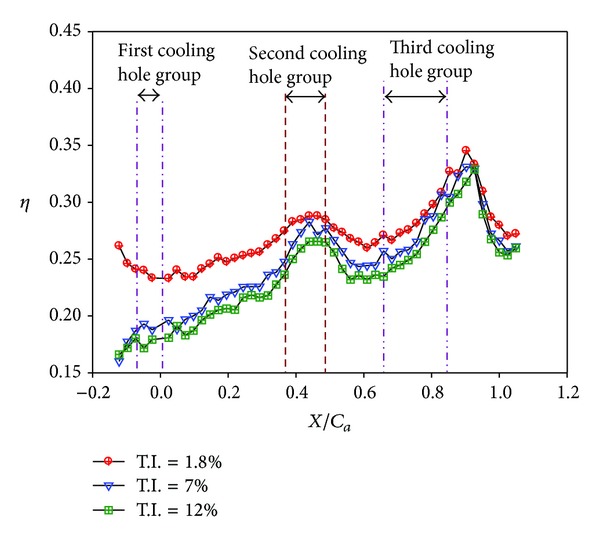
Effect of turbulence intensity on averaged effectiveness for nonstepped endwall (*Re* = 124000, *M* = 2, and *S*/*C* = 0%).

**Figure 20:**
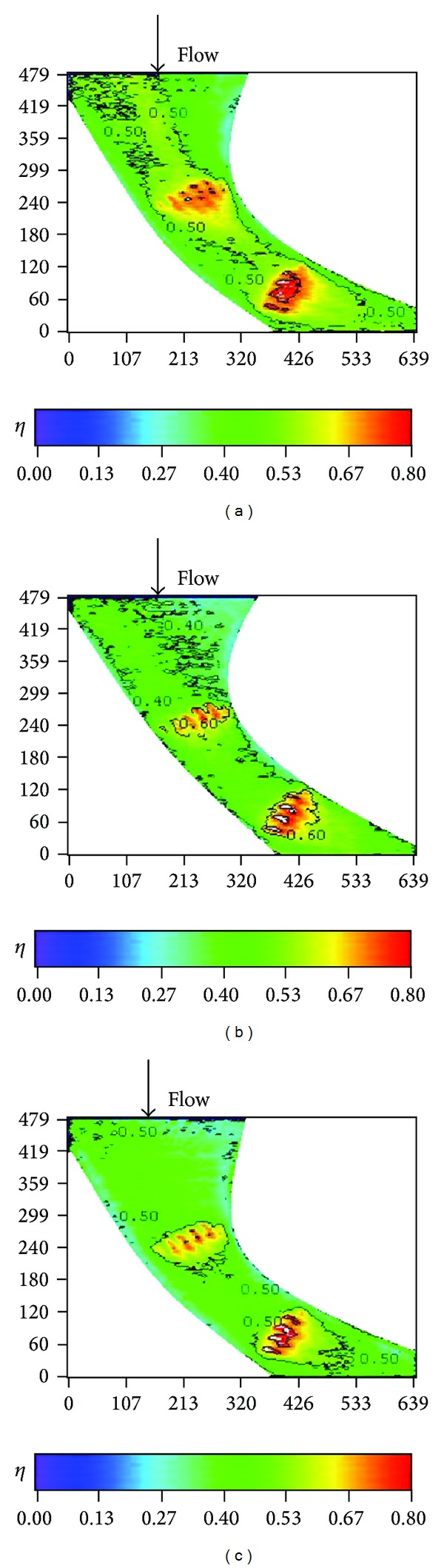
Effect of turbulence intensity on local effectiveness for forward-facing stepped endwall (*Re* = 124000, *M* = 2, and *S*/*C* = 4%). (a) T.I. = 1.8%. (b) T.I. = 7%. (c) T.I. = 12%.

**Figure 21:**
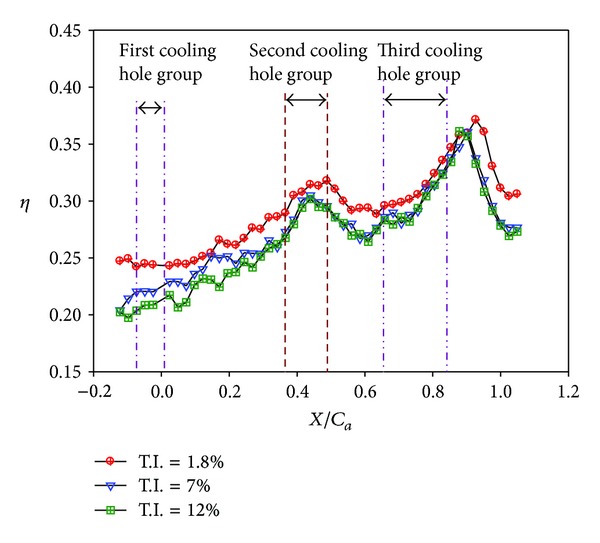
Effect of turbulence intensity on averaged effectiveness for forward-facing stepped endwall (*Re* = 124000, *M* = 2, and *S*/*C* = 4%).

**Figure 22:**
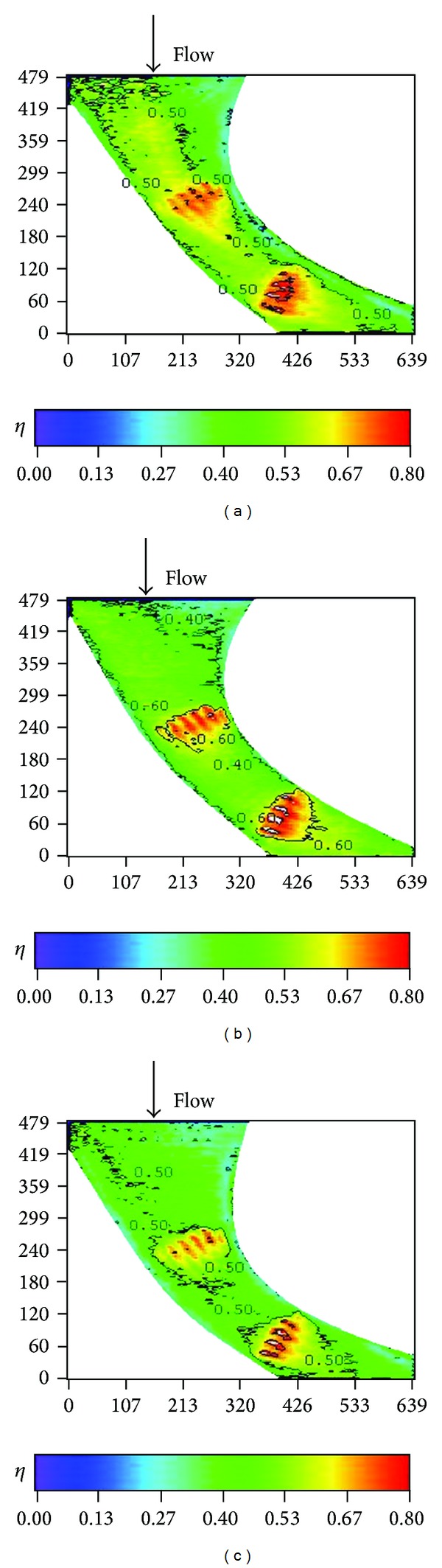
Effect of turbulence intensity on local effectiveness for backward-facing stepped endwall (*Re* = 124000, *M* = 2, and *S*/*C* = −4%). (a) T.I. = 1.8%. (b) T.I. = 7%. (c) T.I. = 12%.

**Figure 23:**
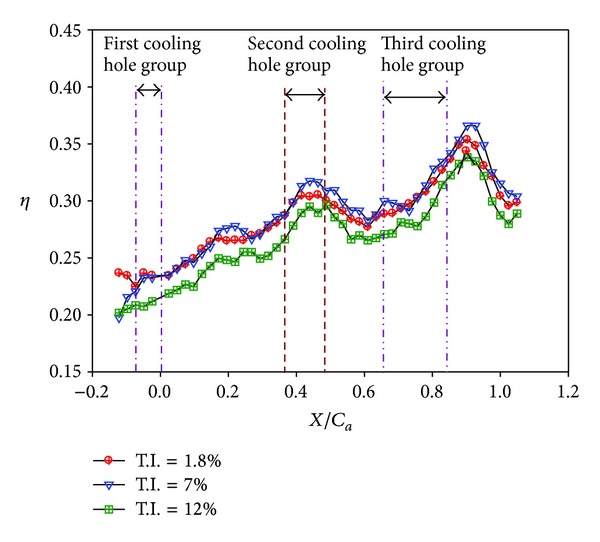
Effect of turbulence intensity on averaged effectiveness for backward-facing stepped endwall (*Re* = 124000, *M* = 2, and *S*/*C* = −4%).

Effects of turbulence intensity on the effectiveness for different endwall conditions are further shown in Figures 18–23 for high blowing ratio (*M* = 2). For the nonstepped endwall cases (Figures 18 and 19), increasing turbulence intensity level obviously decreases the effectiveness, especially in the region from the inlet to the middle of the vane passage. As mentioned above, this is due to thinning of inlet boundary layer, resulting in higher shear and stronger mixing of coolant and main flow. For stepped-endwall cases, regardless of forward-facing step (Figures 20 and 21) or backward-facing step (Figures 22 and 23), the level of influence on effectiveness due to turbulence intensity is reduced, especially for the backward-facing stepped endwall condition. When the main flow passes the step, separation of fluid generates vortices and increases turbulence level near the endwall. If the near-wall turbulence level is higher than or comparable to that of the main flow, the latter becomes less influential. Therefore, the effect of turbulence intensity becomes mild for stepped endwall conditions.

## 5. Conclusions

Thermochromic liquid crystal (TLC) technique with steady-state heat transfer experiments was used to obtain the whole-field film cooling effectiveness at the endwall of a vane passage. Coolant was cross-injected from two-row, paired, inclined holes with nonintersecting centerlines. The following conclusions are drawn from the experimental results of this study.

1. At high turbulence level, the film cooling effectiveness near the leading edge of the vane passage is drastically reduced. This conclusion is common to the combined effects of high turbulence level with all other parameters.
2. At low turbulence intensity, increasing Reynolds number causes the effectiveness to decrease in the vane passage except near the exit. The trend is not monotonic at high turbulence levels when the blowing ratio remains the same.
3. At high turbulence level and low blowing ratio, the cross flow along the endwall induced by the cross-stream pressure gradient increased the effectiveness of the endwall near the pressure surface. Increasing the blowing ratio reduces this benefit.
4. The overall averaged effectiveness for stepped endwall cases is higher than that of the nonstepped endwall case due to redevelopment of the boundary layer. A reversed trend may exist locally at the flow reattachment point for low turbulence case.
5. Increasing turbulence intensity normally decreases the effectiveness. The effect is less important with stepped endwall than nonstepped endwall conditions.
